# Supplementary material for: The immunostimulatory activity of polysaccharides from Glycyrrhiza uralensis
Source: PeerJ. 2020 Jan 29;8:e8294. doi: 10.7717/peerj.8294 (PMC6995267; doi:10.7717/peerj.8294)

Fig 6A

1. P-ERK

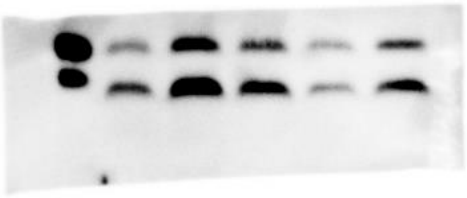

2. ERK

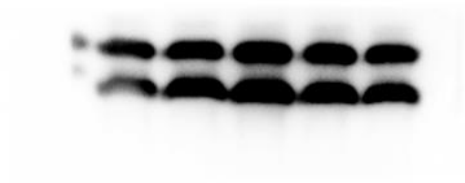

3. P-JNK

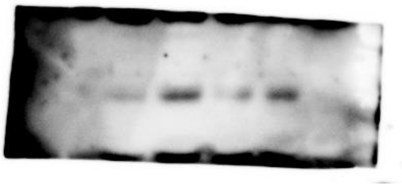

4. JNK

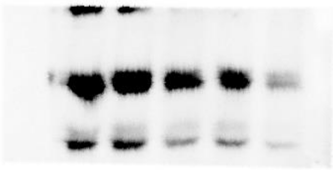

5. p-p38

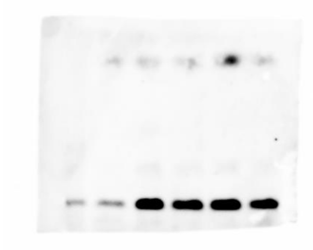

6. p-38

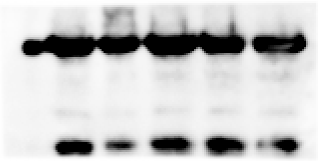

7. p-NF-κBp65

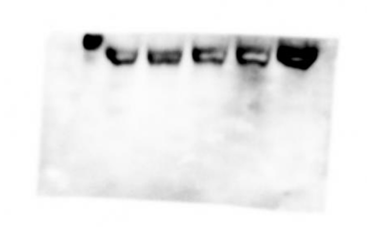

8. NF-κBp65

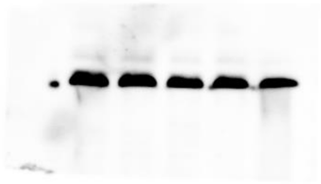

9. P-Ikkα/β

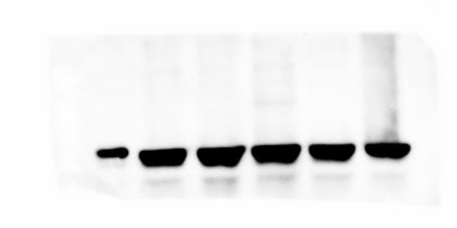

10. Ikkβ

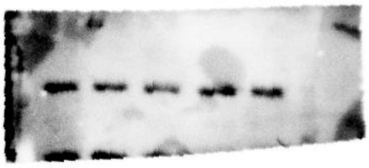

11. Ikkα

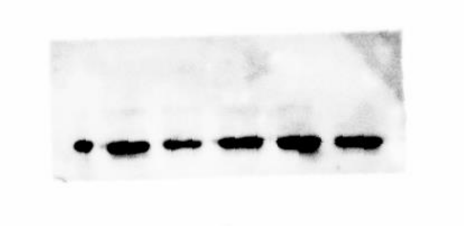

12. p-IκB

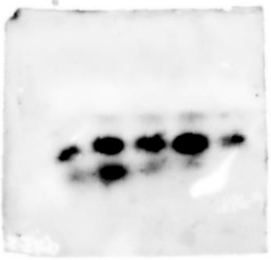

13. IκB

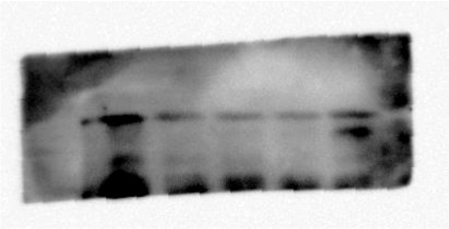

14. β-actin

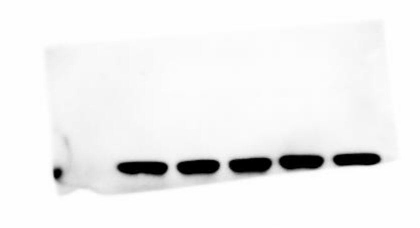

15. NF-κBp65

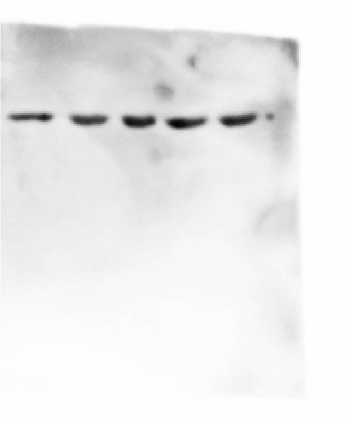

16. Histone

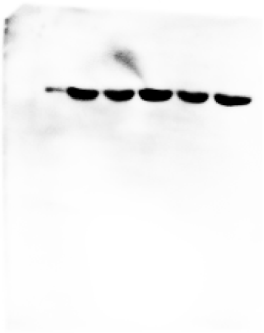

Fig 6B

1. p-ERK

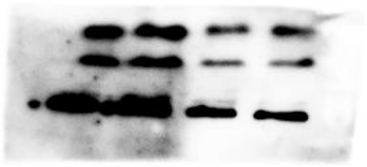

2. ERK

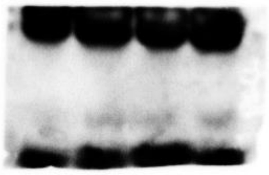

3. p-JNK

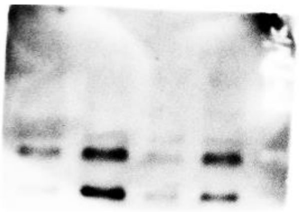

4. JNK

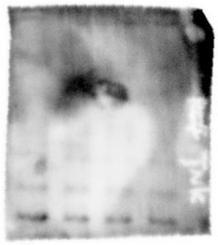

5. p38

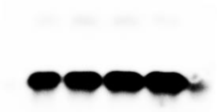

6. p-p38

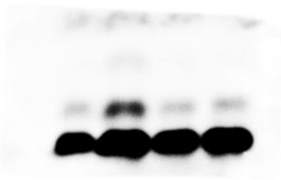

7. p-NK-κBp65

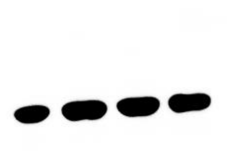

8. NK-κBp65

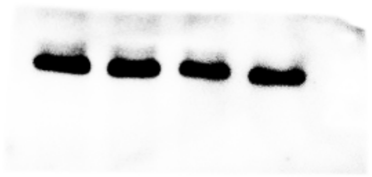

9. p-IKKα/β

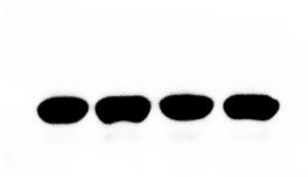

10. IKKβ

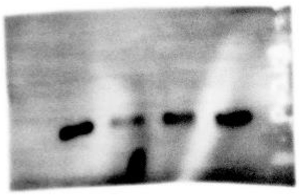

11. IKKα

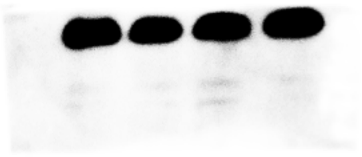

12. p-IκB

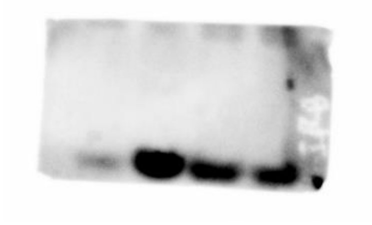

13. IκB

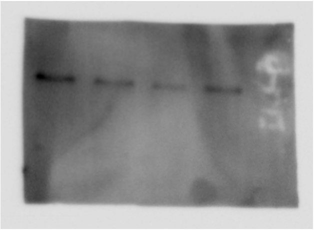

14. β-actin

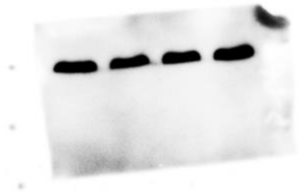

15. NF-κBp65

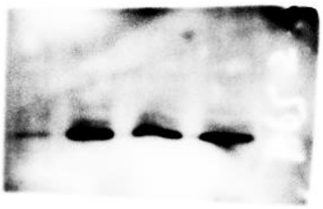

16. Histone

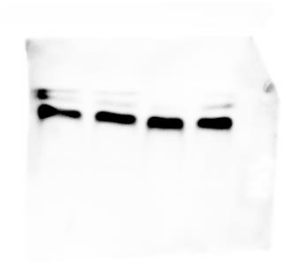

Supplement: Supplemental Information 1 [file peerj-08-8294-s001.pdf]
